# Urinary Metals and Heart Rate Variability: A Cross-Sectional Study of Urban Adults in Wuhan, China

**DOI:** 10.1289/ehp.1307563

**Published:** 2014-10-30

**Authors:** Wei Feng, Xiaosheng He, Mu Chen, Siyun Deng, Gaokun Qiu, Xiaoliang Li, Chuanyao Liu, Jun Li, Qifei Deng, Suli Huang, Tian Wang, Xiayun Dai, Binyao Yang, Jing Yuan, Meian He, Xiaomin Zhang, Weihong Chen, Haidong Kan, Tangchun Wu

**Affiliations:** 1 Department of Occupational and Environmental Health, and; 2 Ministry of Education Key Lab for Environment and Health, School of Public Health, Tongji Medical College, Huazhong University of Science and Technology, Wuhan, China; 3 Department of Nutrition, and; 4 Department of Epidemiology, Harvard School of Public Health, Boston, Massachusetts, USA; 5 School of Public Health, Key Lab of Public Health Safety of the Ministry of Education, Fudan University, Shanghai, China; * These authors contributed equally to this work.

## Abstract

**Background:**

Epidemiological studies have suggested an association between external estimates of exposure to metals in air particles and altered heart rate variability (HRV). However, studies on the association between internal assessments of metals exposure and HRV are limited.

**Objectives:**

The purpose of this study was to examine the potential association between urinary metals and HRV among residents of an urban community in Wuhan, China.

**Methods:**

We performed a cross-sectional analysis of 23 urinary metals and 5-min HRV indices (SDNN, standard deviation of normal-to-normal intervals; r-MSSD, root mean square of successive differences in adjacent normal-to-normal intervals; LF, low frequency; HF, high frequency; TP, total power) using baseline data on 2,004 adult residents of Wuhan.

**Results:**

After adjusting for other metals, creatinine, and other covariates, natural log-transformed urine titanium concentration was positively associated with all HRV indices (all *p* < 0.05). Moreover, we estimated negative associations between cadmium and r-MSSD, LF, HF, and TP; between lead and r-MSSD, HF, and TP; and between iron, copper, and arsenic and HF, SDNN, and LF, respectively, based on models adjusted for other metals, creatinine, and covariates (all *p* < 0.10). Several associations differed according to cardiovascular disease risk factors. For example, negative associations between cadmium and r-MSSD were stronger among participants ≤ 52 years of age (vs. > 52), current smokers (vs. nonsmokers), body mass index < 25 kg/m^2^ (vs. ≥ 25), and among those who were not hypertensive.

**Conclusions:**

Urine concentrations of several metals were associated with HRV parameters in our cross-sectional study population. These findings need replication in other studies with adequate sample sizes.

**Citation:**

Feng W, He X, Chen M, Deng S, Qiu G, Li X, Liu C, Li J, Deng Q, Huang S, Wang T, Dai X, Yang B, Yuan J, He M, Zhang X, Chen W, Kan H, Wu T. 2015. Urinary metals and heart rate variability: a cross-sectional study of urban adults in Wuhan, China. Environ Health Perspect 123:217–222; http://dx.doi.org/10.1289/ehp.1307563

## Introduction

Heavy metal pollution in China has become a serious problem with the rapid industrialization and urbanization of the last two decades, and is therefore a great public health concern (Qu et al. 2012). Exposure to heavy metals has been associated with cardiovascular disease (CVD), even at low and general environmental levels (Mendy et al. 2012; Navas-Acien et al. 2007). One possible mechanism for this association is interference in autonomic modulation of the heart (Park et al. 2006). Heart rate variability (HRV), a physical indicator of cardiac autonomic balance, reflects autonomic modulation of rhythmic heart rate. Accumulating evidence has indicated a link between HRV and estimates of exposure to metals through inhalation of air pollutants (Cavallari et al. 2008; de Hartog et al. 2009; Magari et al. 2002). However, few epidemiological studies have investigated the association between HRV and biomarkers of metal exposures. Jhun et al. (2005) reported that blood cadmium and zinc were associated with altered HRV in 331 Korean participants. Park et al. (2006) observed suggestive association between patella lead and HRV among 129 elderly men with metabolic syndrome from the Normative Aging Study. Furthermore, to our knowledge, no study has investigated the association between biomarkers of metal exposures and HRV in the general Chinese population.

In the present study, we sought to investigate the association between HRV and 23 urinary metals (aluminium, titanium, vanadium, chromium, manganese, iron, cobalt, nickel, copper, zinc, arsenic, selenium, rubidium, strontium, molybdenum, cadmium, tin, antimony, barium, tungsten, thallium, lead, and uranium) in an urban community of Chinese adults. In addition, we explored the potential modification of associations between metals and HRV by model covariates and the Framingham risk score (FRS).

## Materials and Methods

*Study population*. In the present analyses, we used baseline data from a prospective cohort study conducted from April 2011 through May 2011 in Wuhan, China. Participants for the original study were residents of two communities in Wuhan that were selected using a stratified, cluster sampling approach. Residents between 18 and 80 years of age who lived in sampled buildings for > 5 years were invited to participate in a screening evaluation. We excluded residents who were unable to attend clinic visits or had a reduced life expectancy due to a severe illness (e.g., a malignant tumor). Of 3,698 invited community residents, 3,053 (82.6%) individuals agreed to participate in qualitative face-to-face interviews and physical examination, and provided baseline blood and urine samples and questionnaire data. All participants were examined in the morning after an overnight fast. Trained interviewers administered a questionnaire concerning demographic information, occupational and environmental exposures, family and personal diseases, medication use, smoking, alcohol use, diet, and socioeconomic status, as described in detail in our previous work (Song et al. 2014). Qualified physicians performed the study health examination in local community health centers. All participants gave informed consent, and the study protocol was approved by the Ethics and Human Subject Committee of Tongji Medical College.

Participants were excluded from the current analysis for the following reasons: inadequate urine sample volume for metals assays (*n* = 465); bradyarrhythmia (heart rate < 40 beats/min) or tachyarrhythmia (heart rate > 100 beats/min) (*n* = 348), consistent with a previous study (Magari et al. 2002). In addition, we excluded participants who reported a previous diagnosis of CVD (including angina, myocardial infarction, stroke, and other CVDs) (*n* = 150) or kidney disease (*n* = 30) because of potential effects on autonomic function or medication use (Niemelä et al. 1994), or on urinary excretion of metals (Gallieni et al. 1996), respectively. Participants with missing data on covariates were also excluded (*n* = 56). The final study sample consisted of 2,004 individuals.

*Urine collection and storage*. On the morning of each recruitment day, spot urine samples were collected from participants into cleaned conical 50-mL polypropylene tubes, which were then immediately sealed with O-ring screw caps and packed into coolers with frozen ice packs. Samples were sent to the laboratory before 1200 hours, and then stored at –20^o^C and analyzed within 6 months.

*Measurements of HRV*. We measured the HRV indices of participants after urine sample collection. Details of the measurement method have been described previously elsewhere (Li et al. 2012). Briefly, participants were seated for at least 5 min and then fitted with a 3-channel digital Holter monitor (Lifecard CF; Del Mar Reynolds Medical Inc.), which ran at a 1,024 samples/sec sampling rate for 10 min. All measurements were conducted between 0800 and 1200 hours. Data from each Holter assessment were processed using the Impresario and CardioNavigator Plus software (Del Mar Reynolds Medical Inc.). Statistical analyses were based on a single consecutive 5-min segment during the middle portion of each 10-min electrocardiography record (180–480 sec) (Malik et al. 1996). We excluded participants whose heart rates were outside the range of 40–100 beats/min (Magari et al. 2002). We used five measures of HRV including both time and frequency domain outcomes in this analysis. The time domain variables included the standard deviation of all normal R-R intervals (SDNN) and the root mean of square of successive differences between adjacent normal NN intervals (r-MSSD). The frequency domain parameters included low-frequency power (LF, 0.04–0.15 Hz), high-frequency power (HF, 0.15–0.40 Hz), and total power (TP, 0.01–0.40 Hz). In general, 5-min SDNN, r-MSSD, HF, and TP largely reflect vagal tone, whereas LF is related to baroreflex function (Goldstein et al. 2011).

*Determination of urinary metals and creatinine*. We followed a previously published protocol for the measurement of urinary metal concentrations (Heitland and Köster 2006) with minor modifications. We measured the concentrations of urinary metals using an Agilent 7700x inductively coupled plasma mass spectrometer with an octopole-based collision/reaction cell (Agilent Technologies). We used the standard reference materials (SRMs) 2670a (Toxic Elements in Urine) containing low and high concentration levels and 1640a (Trace Elements in Natural Water) as quality control. The two SRMs were purchased from NIST (National Institute of Standards and Technology, Gaithersburg, MD). We used SRM 2670a to verify method accuracy of cobalt, cadmium, antimony, thallium, lead, and uranium at the two concentrations, and of manganese, molybdenum, and selenium at the high concentration. We estimated accuracy by comparing the difference between the certified values available and the measured values with their uncertainty according to the calculation method reported elsewhere (Linsinger 2005). The measurement results by our method were in agreement with the SRM 2670a certified values. Aluminium, vanadium, chromium, nickel, copper, zinc, arsenic, tin, barium, and tungsten in SRM 2670a were not certified; however, the NIST provided reference or information values. The mean results of aluminium, vanadium, chromium, nickel, copper, zinc, arsenic, tin, and barium by the assay agreed within 10.7% of the target value. For tungsten, we determined a concentration of 0.6 μg/L, and the information value provided by NIST was < 1.0 μg/L. Moreover, we used a spiked pooled urine sample (collected randomly from 100 samples) to assess accuracy and precision of the method for determination of titanium, iron, rubidium, and strontium because no certified reference materials were available. The spike recovery values of the four metals were in the range of 78.3%–113.2%. Likewise, we used SRM 1640a to ensure instrument performance, which was certified for all metals except for titanium, rubidium, tin, antimony, and tungsten. The SRM 1640a was analyzed after every 20 samples. The check standards were used to compare elements in case their concentrations were not in agreement with actual concentrations of SRM 1640a. We recalibrated the instrument using multi-element standards and reanalyzed the previous 20 samples if the sample concentrations were significantly different from actual concentrations (Linsinger 2005). The limits of quantification (LOQ) for the urinary metals were in the range 0.0004–0.292 μg/L. We replaced the concentrations of samples below the LOQ with LOQ/2. We used a fully automated clinical chemistry analyzer to measure urine creatinine concentration (Mindray Medical International Ltd.).

*FRS*. We calculated 10-year FRS values for each individual using age, sex, low-density lipoprotein (LDL), high-density lipoprotein (HDL), blood pressure, diabetes, and smoking status, as previously described (Wilson et al. 1998).

*Statistical analyses*. We performed statistical analysis using SPSS (version 12.0; SPSS Inc.). We used Spearman’s rank correlation to assess the association among metals. To improve the normality of measures of HRV and urinary metals, we transformed them by natural logarithm (ln) transformation and examined the distributions of the log-transformed HRV indices using the Kolmogorov–Smirnov test. We then investigated the association between ln-transformed urinary metals (micrograms per liter) and HRV using multivariable linear regression models with adjustment for age, sex, smoking status (never, former, current), pack-years of smoking (among current and former smokers), body mass index (BMI; weight in kilograms divided by height in meters squared), hypertension (systolic blood pressure ≥ 140 mmHg or diastolic blood pressure ≥ 90 mmHg at the time of the study examination, or a previous diagnosis by a physician), hyperlipidemia (total cholesterol > 5.72 mmol/L or triglyceride > 1.70 mmol/L at the study examination, or a previous diagnosis by a physician), diabetes (fasting glucose ≥ 7.0 mmol/L or a previous diagnosis), and urinary creatinine. Because urinary creatinine levels are usually used as an index of standardization for urinary metabolite levels of chemicals to correct for variable dilutions among spot samples, we also investigated HRV associations with ln-transformed urine metal concentrations using creatinine-standardized values (micrograms per millimole creatinine). However, the potential bias of creatinine-standardized urinary chemical concentrations used as independent variables in regression models was well documented (Barr et al. 2005; Schisterman et al. 2005), so we focused primarily on the creatinine-adjusted estimates. We also checked the consistency of estimators by robust chi-square testing based on a previously published method (Cantoni and Ronchetti 2001) if the distributions of log-transformed HRV indices were still skewed (identified by *p* < 0.05 for the Kolmogorov–Smirnov test). We used the false discovery rate (FDR)–corrected *p*-values from 23 hypothesis tests to adjust for multiple testing.We calculated the FDR-adjusted *p*-values by the available spreadsheet software developed by Pike (2011). To investigate the potential impact of multiple metals on each HRV parameter, we constructed full linear regression models that included all metals and covariates (age, sex, smoking status, pack-years, BMI, hypertension, diabetes, and urinary creatinine), and used a backward elimination procedure to retain all metals that predicted the outcome with *p* < 0.10. Because cigarette smoking is an important route of human exposure to heavy metals, we evaluated the difference in urinary metal levels of participants stratified by smoking status (never, former, or current smoker) using univariate analysis of covariance with adjustment for other potential cofounders. We also modeled interaction terms between metals and potential modifiers to examine modification of associations by smoking (nonsmoking or current), sex, age (≤ 52 or > 52 years), BMI (< 25 or ≥ 25 kg/m^2^), hypertension (yes/no), hyperlipidemia (yes/no), and diabetes (yes/no). These models were limited to metals that predicted the outcome with *p* < 0.10 in the multiple-metal models, and included one metal-risk factor interaction at a time, but were adjusted for other metals that were included in the corresponding multiple-metal models. Interaction *p*-values represent the *p*-value for the interaction term. Because the FRS provides a global assessment of cardiovascular risk that incorporates seven traditional risk factors (Wilson et al. 1998), we also evaluated effect modification by FRS, which was categorized as low or high risk according to the median value of 10-year FRS scores (≤ 5% and > 5%, respectively). We defined statistical significance as two-tailed *p* < 0.05 for the single-metal models and *p* < 0.10 for the multiple-metal models.

## Results

The demographic and clinical characteristics as well as HRV measurements of the participants are summarized in Table 1. The median age of the study participants was 51.83 years (5th–95th percentile, 26.83–72.86), which is lower than that of the original study cohort (53.75 years, *p* < 0.001) (see Supplemental Material, Table S1). The study sample included more women (63.8%) than men (36.2%). Most of the population had never smoked (74.3%). The mean smoking index (pack-years) was 26.48 ± 22.67 among former and current smokers.

**Table 1:** Basic characteristics, clinical parameters, and HRV indices of participants in Wuhan city (*n* = 2,004).

Variable	Median (5th–95th percentiles), mean ± SD, or percent
Age (years)	51.83 (26.83–72.86)
Sex
Male	36.2
Female	63.8
Body mass index (kg/m^2^)	23.85 (19.00–29.94)
Smoking
Never	74.3
Former	5.2
Current	20.5
Pack-years	26.48 ± 22.67
Systolic blood pressure (mmHg)	126 (102–167)
Diastolic blood pressure (mmHg)	75 (59–96)
Hypertension	36.0
LDL cholesterol (mmol/L)	3.25 (2.01–4.93)
HDL cholesterol (mmol/L)	1.50 (1.01–2.23)
Triglycerides (mmol/L)	1.17 (0.50–3.67)
Total cholesterol (mmol/L)	4.88 (3.49–6.99)
Fast glucose (mmol/L)	4.82 (3.84–6.90)
Hyperlipidemia	41.7
Diabetes	8.2
FRS, 10-year (%)	5 (1–20)
Urinary creatinine, mmol/L	12.71 (3.56–28.35)
HRV indices
SDNN (msec)	35.00 (18.90–63.13)
r-MSSD (msec)	22.65 (13.20–46.10)
Low frequency (msec^2^)	232.57 (43.11–1137.06)
High frequency (msec^2^)	127.85 (24.34–781.73)
Total power (msec^2^)	855.04 (208.21–3109.79)
Abbreviations: FRS, Framingham risk score; HDL, high-density lipoprotein; LDL, low-density lipoprotein; r-MSSD, square root of the mean squared difference between adjacent normal-to-normal intervals; SDNN, standard deviation of the normal-to-normal intervals.	

The distribution of the 23 urinary metals (standardized and unstandardized for urinary creatinine) is shown in Supplemental Material, Table S2. Tin, tungsten, and lead concentrations were < LOQ in 37.13%, 2.15%, and 5.39% of samples, respectively, and < 0.5% of samples were < LOQ for chromium, cobalt, nickel, and uranium. Concentrations of all other metals were ≥ LOQ in all samples. Spearman’s rank correlation analysis revealed that all 23 metals were positively and significantly associated with each other (all *p* < 0.001) (see Supplemental Material, Table S3).

**Figure 1:**
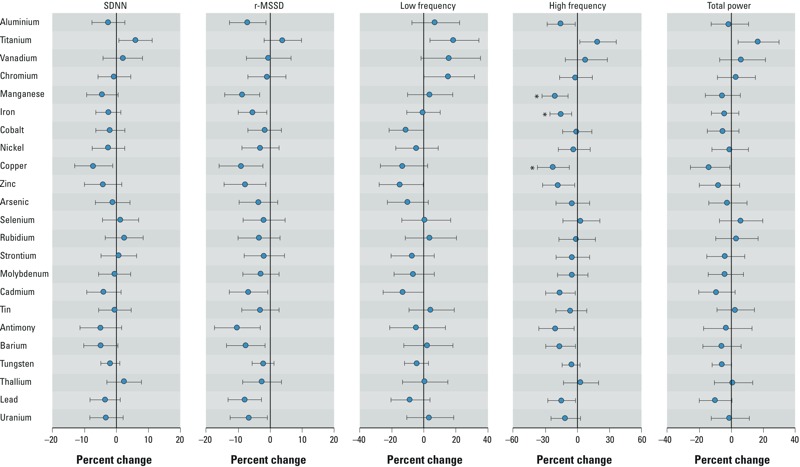
Estimated percent difference (95% CI) in HRV indices with a 10-fold increase in urine metal concentrations based on single-metal linear regression models adjusted for age, sex, smoking status, pack-years, BMI, hypertension, hyperlipidemia, diabetes, and urine creatinine. Abbreviations: FDR, false discovery rate; r-MSSD, square root of the mean squared difference between adjacent normal-to-normal intervals; SDNN, standard deviation of the normal-to-normal intervals. *FDR-adjusted *p* < 0.05.

The distribution of ln-transformed HRV indices is shown in Supplemental Material, Figure S1. All transformed HRV indices were normally distributed except r-MSSD. Robust analyses based on generalized linear regression models showed that findings of significant associations of r-MSSD with urinary aluminium, manganese, iron, copper, zinc, cadmium, antimony, barium, lead, and uranium but not with other urinary metals (data not shown) were consistent with those obtained by multivariate linear regression analysis. Therefore, we reported the estimates of associations between ln-transformed metals and r-MSSD derived using multivariable linear regression models. In the single-metal linear regression models adjusted for age, sex, smoking status, pack-years, BMI, hypertension, hyperlipidemia, diabetes, and urinary creatinine, titanium, and chromium were positively associated with one or more HRV indices, whereas aluminium, manganese, iron, cobalt, copper, zinc, cadmium, antimony, barium, lead, and uranium were negatively related to one or more HRV parameters (all *p* < 0.05) (Figure 1). However, only the associations of manganese, iron, and copper with HF were significant after FDR-adjustment at the 5% alpha level (Figure 1).

Estimates based on models that included multiple metals are shown in Table 2. A 10-fold increase in titanium was associated with 6.84% [95% confidence interval (CI): 1.74, 12.19%], 8.31% (95% CI: 1.98, 15.04%), 25.46% (95% CI: 8.92, 44.51%), 30.78% (95% CI: 11.96, 52.76%), and 24.94% (95% CI: 11.26, 40.30%) increases in SDNN, r-MSSD, LF, HF, and TP, respectively (all *p* < 0.10). In contrast, a 10-fold increase in iron and copper was associated with 12.22% (95% CI: 0.77, 22.34%) and 8.25% (95% CI: 2.12, 14.00%) decreases in HF and SDNN, respectively; a 10-fold increase in arsenic was associated with 19.80% (95% CI: 2.60, 33.96%) decrease in LF; a 10-fold increase in cadmium was associated with 6.58% (95% CI: –0.24, 12.95%), 19.62% (95% CI: 3.65, 32.95%), 18.21% (95% CI: 1.84, 31.84%), and 12.18% (95% CI: –0.59, 23.33%) decreases in r-MSSD, LF, HF, and TP, respectively; a 10-fold increase in lead was associated with 8.31% (95% CI: 2.43, 13.83%), 14.02% (95% CI: –1.20, 26.95%), and 12.41% (95% CI: 1.28, 22.28%) decreases in r-MSSD, HF, and TP, respectively (all *p* < 0.10).

**Table 2:** Estimated percent difference in HRV parameters (95% CI) in association with a 10-fold increase in urine metal concentrations based on multiple-metal models.

Variable	β (95% CIs)	*p*-Value
SDNN
Titanium	6.84 (1.74, 12.19)	0.008
Copper	–8.25 (–14.00, –2.12)	0.009
r-MSSD
Titanium	8.31 (1.98, 15.04)	0.009
Cadmium	–6.58 (–12.95, 0.24)	0.058
Lead	–8.31 (–13.83, –2.43)	0.006
Low frequency
Titanium	25.46 (8.92, 44.51)	0.002
Arsenic	–19.80 (–33.96, –2.60)	0.026
Rubidium	22.69 (–1.42, 52.70)	0.067
Cadmium	–19.62 (–32.95, –3.65)	0.018
High frequency
Titanium	30.78 (11.96, 52.76)	0.001
Iron	–12.22 (–22.34, –0.77)	0.037
Cadmium	–18.21 (–31.84, –1.84)	0.031
Lead	–14.02 (–26.95, 1.20)	0.069
Total power
Titanium	24.94 (11.26, 40.30)	< 0.001
Cadmium	–12.18 (–23.33, 0.59)	0.061
Lead	–12.41 (–22.28, –1.28)	0.030
Abbreviations: r-MSSD, square root of the mean squared difference between adjacent normal-to-normal intervals; SDNN, standard deviation of the normal-to-normal intervals. Metals (natural log-transformed) were selected by backward elimination in multivariate linear regression models (alpha = 0.10) with adjustment for age, sex, smoking status, pack-year, BMI, hypertension, hyperlipidemia, diabetes, and urinary creatinine, respectively.		

The results of the association between single metals and HRV based on the models of creatinine-standardized metals are depicted in Supplemental Material, Figure S2. Titanium and chromium were positively associated with one or more HRV indices, which were consistent with the findings obtained by the models adjusted for creatinine as a covariate. However, the significant associations of vanadium and thallium with HRV identified by the creatinine-standardized single-metal models were not observed in the single or multiple metal models adjusted for creatinine as a covariate. Aluminium and manganese were not related to HRV based on creatinine-standardized single-metal analysis; however, we observed divergent association of the metals with HRV in the output of creatinine-standardized multiple-metal and unstandardized single-metal models (see Supplemental Material, Table S4). Interestingly, we found significant associations of titanium, arsenic, rubidium, cadmium, and lead with one or more HRV parameters in the creatinine-standardized multiple-metal models (see Supplemental Material, Table S4), which were in accordance with the estimate based on the multiple-metals models adjusted for creatinine as a covariate, although the established associations of selenium and strontium with HRV by the analysis of multiple metal standardized for creatinine were not observed in the single- or multiple-metal models that included creatinine as a covariate.

Furthermore, we found that urinary levels of cadmium were higher in current smokers than in former smokers or in those who had never smoked when adjusted for covariates and multiple testing (data not shown). Age, smoking status, BMI, hypertension, and diabetes modified the association between multiple metals and HRV based on adjusted models that included interaction terms of one metal × potential modifiers (see Supplemental Material, Tables S5–S7). For example, negative associations of cadmium with r-MSSD, LF, and TP were stronger among participants ≤ 52 than in those > 52 years of age, and among current smokers than in nonsmokers; and negative associations with r-MSSD were stronger among those with lower BMI (< 25 vs. ≥ 25 kg/m^2^) and in nonhypertensive versus hypertensive participants (all *p* for interaction < 0.10). Negative associations of HF with iron, SDNN with copper, and LF with arsenic, were stronger among diabetics than nondiabetics (all *p* for interaction < 0.10). The negative association between iron and HF was stronger among those with a high-risk FRS compared with a low-risk FRS (*p* for interaction < 0.10).

## Discussion

We estimated heterogeneous associations of increased or decreased HRV indices with aluminium, titanium, chromium manganese, iron, cobalt, copper, zinc, cadmium, antimony, barium, lead, and uranium based on single-metal models. Interpretation of associations with individual metals was limited by interrelated issues, such as covariation among pollutants, the possibility of complex interactions among pollutants, and power constraints (Tolbert et al. 2007). However, associations of titanium, iron, copper, cadmium, and lead with one or more HRV parameters were consistent between single- and multiple-metal analyses. In contrast, LF was associated with arsenic and rubidium in the multiple-metal model only. To the best of our knowledge, this is the first study using urinary data to investigate the effects of exposure to metals on cardiac autonomic function in a community-based population.

Although some elements (copper, zinc) participate in the regulation of physiological functions at limited levels, others such as cadmium and lead have been shown to be toxic even at very low concentration (Mendy et al. 2012). Metals enter the human body mainly by inhalation and ingestion and then accumulate in bone and in tissues and organs such as liver, kidney, and muscle, with half-lives up to several days or decades (Quandt et al. 2010). Urine is the main route of excretion of metals, and urine concentrations of most metals reflect previous exposure within a few hours or days, except for cadmium, for which urinary outputs reflect cumulative exposure (Tellez-Plaza et al. 2012). Titanium dioxide has been classified by the International Agency for Research on Cancer (IARC) as possibly carcinogenic to humans (IARC 2010). However, no previous epidemiological study was conducted to investigate the association between titanium and risk of cardiovascular disease. In the present study, titanium was associated with higher values of multiple HRV indices based on single- and multiple-metal models. Higher values for time and frequency domain indices may indicate that titanium increases parasympathetic (vagal) influence and baroreceptor activity, suggesting a possible beneficial (rather than adverse) effect, though noncausal explanations for this and other findings cannot be ruled out. Epidemiologic studies have reported associations between HRV and exposure to transition metals in particulate matter (PM), such as vanadium, chromium, manganese, iron, and nickel (Cavallari et al. 2008; Magari et al. 2002; Wu CF et al. 2012; Wu S et al. 2011). We did not find that urinary nickel was associated with any HRV indices. Urinary outputs of nickel mainly reflect the exposure to soluble compounds, and the toxicity of nickel may be associated with its interference with the physiological processes of nutrient elements such as manganese, calcium, and zinc (Coogan et al. 1989). Nevertheless, in the present study, HRV indices were negatively associated with urinary manganese in single-metal models, and with iron and copper in both single- and multiple-metal models. Additional research is needed to determine whether associations of HRV parameters and transition metals in urine reflect effects of exposure to metals in PM on cardiac autonomic function. HRV was not associated with urine vanadium or chromium in our study population. However, we found that urinary vanadium was associated with HRV in both single- and multiple-metal models, and chromium was related to HRV in single-metal models when we re-analyzed the data of urinary metals standardized for creatinine. Interestingly, we found consistent associations of titanium, arsenic, cadmium, and lead with one or more HRV parameters in both creatinine-standardized and unstandardized multiple-metal models. Traditionally, metabolites of foreign substances in spot urine divided by urinary creatinine for variable dilutions were usually used to assess exposure. However, evidence has suggested that potential bias may be introduced if creatinine-standardized urinary chemical concentrations are used as independent variables in regression models (Barr et al. 2005; Schisterman et al. 2005). Therefore, in the present analysis, the urinary metal and creatinine concentrations were treated separately as two independent variables in the multiple linear regressions. Nonetheless, the reasons for the difference in the results obtained from the analyses of metal concentration standardized and unstandardized for creatinine remain unclear and warrant further investigation.

Cadmium and lead are both environmental toxicants that have been associated with adverse cardiovascular events, such as hypertension and coronary heart disease (CHD), in epidemiologic studies (Mordukhovich et al. 2012; Navas-Acien et al. 2007; Tellez-Plaza et al. 2013). Consistent with the previous epidemiological studies on blood cadmium (Jhun et al. 2005) and patella lead (Park et al. 2006), urinary cadmium and lead both were associated with reduced HRV based on single- and multiple-metal models.

Arsenic is a potent human carcinogen. Exposure to arsenic, particularly inorganic arsenic, poses a potential health risk to human (Smith et al. 1992). Urinary arsenic reflects mainly exposure to inorganic arsenic (Ahmed et al. 2014). Increasing numbers of studies have suggested that urinary arsenic is correlated with CVD (Navas-Acien et al. 2005). In the present study, no association between urinary arsenic and HRV was observed in individual metal models. However, urinary arsenic was associated with decreased LF based on multiple-metal models. Similarly, a previous work reported that arsenic levels in drinking water were associated with electrocardiographic (ECG) abnormalities in residents of the Ba Men region of Inner Mongolia (Mumford et al. 2007).

Rubidium, which is believed to have a low degree of toxicity (Berry et al. 1972), was associated with LF based on a multiple-metal model (*p* = 0.067). To our knowledge, no previous study has examined the relationship between rubidium and cardiac autonomic function. The observed associations of arsenic and rubidium with HRV parameters based on multiple-metal models should be interpreted with caution. Arsenic and rubidium might be predictors or surrogate measures of other metals that affect baroreflex function. It is also possible that associations with arsenic and rubidium reflect interactions among multiple metals, dependent measurement errors, or residual confounding (Sun et al. 2013; Tolbert et al. 2007; Zeger et al. 2000).

Although underlying biological mechanisms that might link metal exposures to cardiac autonomic dysfunction are unidentified, neurocardiac effects mediated through oxidative stress, enzymatic inhibition, and impaired antioxidant metabolism may play a role (Hearse 1991; Jomova and Valko 2011; Rhoden et al. 2005). Transition and toxic metals like manganese, iron, copper, and lead undergo redox cycling reactions and possess the ability to produce reactive oxygen species (ROS) such as superoxide anion radical and nitric oxide (Jomova and Valko 2011). Redox-inert metals such as arsenic and cadmium are unable to generate free radicals directly; however, enhanced ROS generation can occur via suppression of free radical scavengers (such as glutathione), by inhibition of detoxifying enzymes and perhaps through other indirect mechanisms involving the displacement of Fenton metals from proteins (Waisberg et al. 2003).

Cigarette smoke contains toxic and cancer-causing chemicals such as nicotine and heavy metals (Chiba and Masironi 1992). Consistent with previous studies (Kim et al. 2010; Mortensen et al. 2011), our data indicated that urinary cadmium was higher in current smokers than in ex-smokers and in those who had never smoked. Stratified analyses indicated that age, smoking status, BMI, and hypertension modified the association between cadmium and HRV. For example, the negative association of cadmium with HRV was stronger in current smokers than that among nonsmokers. Potentially, cadmium could have synergistic effects with other toxicants in tobacco such as carbon monoxide, nitrogen oxides, and hydrogen cyanide (Navas-Acien et al. 2004). We also found that negative associations between copper and SDNN, arsenic and LF, and iron and HF were stronger among diabetic than in nondiabetic participants. The FRS is one of the standard tools used to predict the incidence of CHD (Wilson et al. 1998). A recent study suggested a significant interaction effect of FRS with polycyclic aromatic hydrocarbons on HRV (Feng et al. 2014). Our analysis indicated that iron may have a stronger negative association with HF among individuals at high versus low CHD risk based on the FRS. In theory, combined effects of oxidative stress due to metals exposures and diabetes or other CHD risk factors could have a synergistic influence on cardiovascular autonomic function (Park et al. 2006).

Several limitations to our study should be considered. First, spot urine testing was used to assess exposure to metals, which may have introduced measurement error due to individual variability in metal and creatinine excretion throughout the day. Second, there may be dependent measurement error due to multiple metals measured in the same urine sample by the same assay, therefore resulting in potentially misleading findings (Zeger et al. 2000). Third, urinary concentrations of some metals may not reliably reflect environmental exposure, and may therefore lead to possible exposure misclassification. Future studies should measure these metals in the blood to examine the associations. Finally, the current findings of both positive and negative associations between 23 urinary metals and five HRV parameters based on an exploratory cross-sectional analysis need further investigation in other studies.

## Conclusions

Results of this study suggest that exposure to metals may contribute to cardiac autonomic dysregulation. However, we cannot rule out chance or bias due to dependent measurement error or model misspecification, and underlying mechanisms to explain associations, including an unexpected association between titanium and increased HRV, are not known. Therefore, our preliminary findings need to be replicated in other large study populations.

## Supplemental Material

(1.3 MB) PDF
