# Supplementary material for: Urinary Metals and Heart Rate Variability: A Cross-Sectional Study of Urban Adults in Wuhan, China
Source: Environ Health Perspect. 2014 Oct 30;123(3):217–22. doi: 10.1289/ehp.1307563 (PMC4348740; doi:10.1289/ehp.1307563)

Environmental Health Perspectives doi:10.1289/ehp.1307563

Supplemental Material

Urinary Metals and Heart Rate Variability: A Cross-Sectional Study of Urban Adults in Wuhan, China

Wei Feng, Xiaosheng He, Mu Chen, Siyun Deng, Gaokun Qiu, Xiaoliang Li, Chuanyao Liu, Jun Li, Qifei Deng, Suli Huang, Tian Wang, Xiayun Dai, Binyao Yang, Jing Yuan, Meian He, Xiaomin Zhang, Weihong Chen, Haidong Kan, and Tangchun Wu

Table S1. Basic characteristics and HRV indices of original cohort population and source population in Wuhan city.

Variable (unit)	Original cohort population (n=3053)	Source population (n=2004)	*P*
Age (year)	53.75 (28.67-74.86)	51.83 (26.83-72.86)	< 0.001
Gender: male/female (%)	35.6/64.6	36.2/63.8	0.593
Body mass index (kg/m^2)	23.92 (19.05-30.12)	23.85 (19.00-29.94)	0.801
Smokers: never/former/current (%)	74.7/5.7/19.6	74.3/5.2/20.5	0.590
Pack years, mean ± SD	26.80 ± 22.81	26.48 ± 22.67	0.795
HRV indices			
SDNN (msec)	34.60 (18.90-63.38)	35.00 (18.90-63.13)	0.320
r-MSSD (msec)	22.50 (13.20-46.88)	22.65 (13.20-46.10)	0.626
Low frequency (ms^2)	219.12 (39.85-1134.55)	232.57 (43.11-1137.06)	0.086
High frequency (ms^2)	125.93 (22.26-801.44)	127.85 (24.34-781.73)	0.513
Total power (ms^2)	819.36 (204.86-3250.73)	855.04 (208.21-3109.79)	0.168

Abbreviations: SDNN, standard deviation of the normal-to-normal intervals; r-MSSD, square root of the mean squared difference between adjacent normal-to-normal intervals. Data were presented as median (5–95% percentiles). Chi-square test and Mann Whitney U test were performed for comparison of the categorical and continuous variables respectively.

Table S2. The distributions of urinary metals in the community-dwelling population (n=2004).

Urinary metals	Geometric mean	Percentile 5th	Percentile 25th	Percentile 50th	Percentile 75th	Percentile 95th	N (%) < LOQ
**Unstandardized for urinary creatinine (μg/L)**							
Aluminium	33.80	12.77	21.99	32.02	49.15	113.05	0 (0.00)
Titanium	41.96	10.68	25.23	44.42	73.53	140.60	0 (0.00)
Vanadium	0.51	0.19	0.35	0.50	0.75	1.34	0 (0.00)
Chromium	1.45	0.50	0.93	1.46	2.24	4.35	3 (0.15)
Manganese	2.56	0.88	1.67	2.53	3.84	7.66	0 (0.00)
Iron	85.27	22.48	46.53	80.08	146.72	378.07	0 (0.00)
Cobalt	0.27	0.08	0.16	0.25	0.43	1.28	2 (0.10)
Nickel	2.36	0.76	1.50	2.35	3.65	7.90	5 (0.25)
Copper	7.70	2.85	5.26	7.59	11.23	20.62	0 (0.00)
Zinc	276.94	84.09	171.15	278.92	446.40	912.32	0 (0.00)
Arsenic	28.02	6.50	17.25	29.51	48.50	92.37	0 (0.00)
Selenium	7.28	1.93	4.51	7.65	12.19	23.54	0 (0.00)
Rubidium	1856.88	431.25	1212.60	2067.03	3191.14	5236.96	0 (0.00)
Strontium	117.35	35.10	76.79	125.40	186.47	319.81	0 (0.00)
Molybdenum	45.37	10.49	27.35	47.46	80.51	159.89	0 (0.00)
Cadmium	0.87	0.23	0.53	0.91	1.48	2.97	0 (0.00)
Tin	0.25	< LOQ	< LOQ	0.28	0.41	0.76	744 (37.13)
Antimony	0.16	0.06	0.11	0.16	0.24	0.42	0 (0.00)
Barium	4.01	1.53	2.64	3.91	5.92	11.29	0 (0.00)
Tungsten	0.12	0.03	0.07	0.12	0.22	0.75	43 (2.15)
Thallium	0.52	0.13	0.34	0.57	0.89	1.52	0 (0.00)
Lead	3.08	0.45	2.19	3.31	4.67	8.94	108 (5.39)
Uranium	0.03	0.01	0.02	0.03	0.05	0.08	9 (0.45)
**Urinary creatinine-standardized (μg/mmol creatinine)**							
Aluminium	2.90	0.90	1.65	2.63	4.61	13.74	0 (0.00)
Titanium	3.60	1.07	2.16	3.74	6.11	10.57	0 (0.00)
Vanadium	0.04	0.02	0.03	0.04	0.07	0.13	0 (0.00)
Chromium	0.12	0.04	0.07	0.12	0.21	0.49	3 (0.15)
Manganese	0.22	0.06	0.13	0.21	0.36	0.96	0 (0.00)
Iron	7.31	1.78	3.75	6.76	13.10	38.09	0 (0.00)
Cobalt	0.02	0.01	0.01	0.02	0.04	0.11	2 (0.10)
Nickel	0.20	0.07	0.12	0.19	0.32	0.72	5 (0.25)
Copper	0.66	0.28	0.44	0.62	0.91	1.85	0 (0.00)
Zinc	23.75	8.70	16.24	23.43	35.23	64.32	0 (0.00)
Arsenic	2.40	0.89	1.68	2.42	3.45	6.43	0 (0.00)
Selenium	0.62	0.25	0.43	0.62	0.91	1.50	0 (0.00)
Rubidium	159.24	61.70	114.27	164.71	230.54	358.77	0 (0.00)
Strontium	10.06	3.26	6.66	10.55	15.63	28.35	0 (0.00)
Molybdenum	3.89	1.29	2.58	3.99	5.95	10.87	0 (0.00)
Cadmium	0.08	0.03	0.05	0.08	0.11	0.22	0 (0.00)
Tin	0.02	< LOQ	< LOQ	0.02	0.03	0.08	744 (37.13)
Antimony	0.01	0.01	0.01	0.01	0.02	0.04	0 (0.00)
Barium	0.34	0.11	0.20	0.33	0.56	1.37	0 (0.00)
Tungsten	0.01	0.00	0.01	0.01	0.02	0.06	43 (2.15)
Thallium	0.05	0.02	0.03	0.05	0.07	0.12	0 (0.00)
Lead	0.26	0.08	0.18	0.26	0.41	0.87	108 (5.39)
Uranium	0.00	0.00	0.00	0.00	0.00	0.01	9 (0.45)

Abbreviations: LOQ, limits of quantification.

Table S3. Associations between urinary metals (Spearman's rank correlation coefficients, all $p < 0.001$).

	Al	Ti	V	Cr	Mn	Fe	Co	Ni	Cu	Zn	As	Se	Rb	Sr	Mo	Cd	Sn	Sb	Ba	W	Tl	Pb	U
**Al**	1.00	0.19	0.29	0.34	0.69	0.34	0.25	0.27	0.29	0.25	0.14	0.19	0.16	0.20	0.15	0.21	0.37	0.37	0.51	0.31	0.19	0.46	0.42
**Ti**		1.00	0.65	0.52	0.09	0.10	0.36	0.34	0.29	0.30	0.46	0.70	0.50	0.37	0.39	0.40	0.44	0.36	0.14	0.27	0.48	0.36	0.24
**V**			1.00	0.79	0.28	0.23	0.38	0.39	0.45	0.33	0.45	0.58	0.49	0.39	0.42	0.38	0.45	0.46	0.31	0.34	0.49	0.48	0.37
**Cr**				1.00	0.34	0.23	0.26	0.35	0.36	0.20	0.23	0.37	0.27	0.21	0.20	0.21	0.37	0.28	0.32	0.21	0.31	0.41	0.26
**Mn**					1.00	0.70	0.32	0.30	0.34	0.32	0.20	0.16	0.19	0.17	0.19	0.22	0.35	0.38	0.46	0.28	0.17	0.44	0.35
**Fe**						1.00	0.32	0.23	0.30	0.34	0.29	0.23	0.26	0.17	0.26	0.25	0.28	0.32	0.24	0.21	0.19	0.30	0.22
**Co**							1.00	0.58	0.43	0.41	0.52	0.50	0.52	0.41	0.52	0.54	0.41	0.49	0.21	0.36	0.50	0.40	0.25
**Ni**								1.00	0.54	0.45	0.45	0.44	0.40	0.30	0.45	0.46	0.34	0.50	0.23	0.32	0.33	0.39	0.32
**Cu**									1.00	0.58	0.54	0.46	0.47	0.32	0.49	0.54	0.37	0.58	0.30	0.36	0.40	0.45	0.48
**Zn**										1.00	0.59	0.57	0.51	0.40	0.54	0.53	0.37	0.56	0.22	0.38	0.41	0.43	0.39
**As**											1.00	0.77	0.76	0.55	0.74	0.64	0.44	0.66	0.16	0.43	0.61	0.41	0.42
**Se**												1.00	0.78	0.54	0.67	0.60	0.54	0.62	0.17	0.45	0.65	0.45	0.38
**Rb**													1.00	0.42	0.63	0.65	0.46	0.60	0.11	0.37	0.83	0.42	0.35
**Sr**														1.00	0.51	0.44	0.37	0.48	0.47	0.42	0.48	0.47	0.36
**Mo**															1.00	0.57	0.40	0.62	0.13	0.50	0.53	0.40	0.39
**Cd**																1.00	0.40	0.56	0.20	0.36	0.56	0.44	0.41
**Sn**																	1.00	0.53	0.27	0.41	0.43	0.49	0.37
**Sb**																		1.00	0.34	0.55	0.47	0.53	0.63
**Ba**																			1.00	0.26	0.21	0.41	0.43
**W**																				1.00	0.36	0.41	0.44
**Tl**																					1.00	0.47	0.30
**Pb**																						1.00	0.40
**U**																							1.00

Abbreviations: Al, Aluminium; Ti, Titanium; V, Vanadium; Cr, Chromium; Mn, Manganese; Fe, Iron; Co, Cobalt; Ni, Nickel; Cu, Copper; Zn, Zinc; As, Arsenic; Se, Selenium; Rb, Rubidium; Sr, Strontium; Mo, Molybdenum; Cd, Cadmium; Sn, Tin; Sb, Antimony; Ba, Barium; W, Tungsten; Tl, Thallium; Pb, Lead; U, Uranium.

Table S4. Estimated percent difference in HRV parameters (95% CI) in association with a 10-fold increase in creatinine-standardized urine metal concentrations based on multiple-metal models.

**Variable**	**β (95% CIs)**	***p***
**SDNN**		
Titanium	12.24 (5.50, 19.41)	< 0.001
Selenium	-9.73 (-18.07, -0.54)	0.038
Rubidium	8.06 (-0.86, 17.79)	0.078
Strontium	5.44 (-0.62, 11.87)	0.079
Cadmium	-7.88 (-14.12, -1.19)	0.022
**r-MSSD**		
Titanium	13.95 (5.96, 22.53)	< 0.001
Selenium	-8.67 (-16.87, 0.34)	0.059
Strontium	7.18 (-0.31, 15.24)	0.061
Lead	-6.41 (-12.04, -0.43)	0.036
**Low frequency**		
Titanium	31.61 (14.37, 51.44)	< 0.001
Manganese	16.41 (2.28, 32.51)	0.021
Arsenic	-21.24 (-35.07, -4.46)	0.015
Rubidium	25.01 (-0.16, 56.53)	0.052
Cadmium	-18.61 (-32.45, -1.94)	0.030
Lead	-13.49 (-25.66, 0.68)	0.061
**High frequency**		
Titanium	28.46 (6.79, 54.53)	0.008
Vanadium	19.78 (-2.02, 46.44)	0.078
Cadmium	-21.11 (-34.76, -4.61)	0.014
**Total power**		
Aluminium	11.92 (-0.04, 25.32)	0.051
Titanium	28.09 (14.02, 43.90)	< 0.001
Cadmium	-13.45 (-24.98, -0.15)	0.048
Lead	-11.14 (-21.79, 0.96)	0.070

Abbreviations: SDNN, standard deviation of the normal-to-normal intervals; r-MSSD, square root of the mean squared difference between adjacent normal-to-normal intervals.

Metals (natural log-transformed) were selected by backward elimination in multivariate linear regression models (alpha = 0.10) with adjustment for age, gender, smoking status, pack year, BMI, hypertension, hyperlipidemia and diabetes, respectively.

Table S5. Percent difference (95% CI) in HRV parameters according to age, gender and smoking status.

Variable	Age (≤ 52 yrs, n=1003)	Age (> 52 yrs, n=1001)	*p* for interaction	Male (n=725)	Female (n=1279)	*p* for interaction	Non-smokers (n=1594)*	Current smokers (n=410)	*p* for interaction
**SDNN**									
Titanium	2.49 (-4.05, 9.48)	9.93 (1.92, 18.56)	0.417	10.52 (1.38, 20.48)	5.11 (-0.96, 11.54)	0.233	6.67 (0.97, 12.69)	7.24 (-3.88, 19.64)	0.179
Copper	-5.40 (-13.69, 3.69)	-11.61 (-19.68, -2.73)	0.518	-7.52 (-17.76, 4.00)	-9.00 (-15.84, -1.61)	0.854	-8.70 (-14.96, -1.98)	-10.04 (-23.25, 5.45)	0.101
**r-MSSD**									
Titanium	5.02 (-2.73, 13.39)	10.80 (0.88, 21.69)	0.363	12.49 (1.63, 24.51)	5.88 (-1.79, 14.16)	0.305	7.42 (0.21, 15.16)	9.24 (-3.16, 23.43)	0.542
Cadmium	-14.08 (-21.23, -6.27)	4.20 (-7.13, 16.92)	0.041	-0.71 (-12.98, 13.29)	-8.99 (-16.36, -0.97)	0.273	-5.63 (-12.81, 2.14)	-10.88 (-23.89, 4.36)	0.074
Lead	-0.45 (-7.97, 7.67)	-16.48 (-24.23, -7.95)	0.146	-12.36 (-21.38, -2.31)	-5.90 (-12.82, 1.57)	0.665	-7.30 (-13.49, -0.67)	-14.08 (-25.89, -0.40)	0.888
**Low frequency**									
Titanium	15.08 (-4.88, 39.23)	37.55 (10.10, 71.83)	0.163	30.20 (3.37, 63.98)	22.43 (2.22, 46.64)	0.619	29.93 (10.24, 53.15)	11.83 (-15.52, 48.04)	0.460
Arsenic	-34.58 (-49.62, -15.04)	-18.67 (-40.00, 10.22)	0.239	-19.47 (-42.26, 12.32)	-19.22 (-36.62, 2.96)	0.828	-21.51 (-36.97, -2.27)	-14.15 (-43.95, 31.47)	0.530
Rubidium	48.25 (11.85, 96.49)	10.39 (-23.15, 58.56)	0.616	49.89 (3.38, 117.33)	8.55 (-17.27, 42.43)	0.640	13.06 (-11.69, 44.75)	61.35 (0.09, 160.10)	0.536
Cadmium	-32.18 (-46.37, -14.24)	-8.11 (-32.30, 24.71)	0.024	-38.45 (-55.84, -14.22)	-8.36 (-26.42, 14.13)	0.244	-11.71 (-27.89, 8.11)	-45.99 (-64.00, -18.97)	0.055
**High frequency**									
Titanium	21.18 (-1.78, 49.49)	37.97 (9.24, 74.25)	0.584	32.76 (2.31, 72.28)	29.37 (6.48, 57.16)	0.901	31.40 (9.88, 57.12)	27.00 (-7.74, 74.82)	0.546
Iron	-10.11 (-23.86, 6.12)	-15.14 (-29.32, 1.87)	0.417	-8.88 (-27.67, 14.79)	-13.89 (-25.45, -0.53)	0.625	-12.46 (-23.56, 0.25)	-9.50 (-32.38, 21.12)	0.520
Cadmium	-33.43 (-47.60, -15.42)	0.53 (-24.54, 33.92)	0.152	-7.35 (-34.13, 30.34)	-23.92 (-38.86, -5.32)	0.304	-17.54 (-32.73, 1.08)	-22.21 (-48.89, 18.38)	0.533
Lead	6.81 (-14.20, 32.96)	-32.59 (-47.29, -13.80)	0.067	-25.97 (-44.20, -1.77)	-6.22 (-23.33, 14.71)	0.378	-12.90 (-27.30, 4.36)	-22.48 (-47.79, 15.10)	0.780
**Total power**									
Titanium	14.31 (-2.08, 33.44)	35.94 (13.52, 62.79)	0.129	33.68 (9.39, 63.35)	19.46 (3.62, 37.73)	0.533	26.03 (10.48, 43.79)	19.24 (-7.02, 52.91)	0.823
Cadmium	-30.42 (-41.62, -17.07)	8.37 (-13.15, 35.22)	0.006	-20.79 (-38.95, 2.77)	-7.17 (-20.87, 8.91)	0.412	-9.22 (-21.85, 5.46)	-26.24 (-46.63, 1.94)	0.049
Lead	-3.01 (-17.21, 13.64)	-24.30 (-37.21, -8.73)	0.555	-15.56 (-31.86, 4.64)	-10.91 (-22.89, 2.94)	0.659	-11.18 (-22.08, 1.24)	-22.44 (-42.72, 5.02)	0.465

Abbreviations: SDNN, standard deviation of the normal-to-normal intervals; r-MSSD, square root of the mean squared difference between adjacent normal-to-normal intervals. Each interaction model included one metal-risk factor interaction term and covariates (age, gender, smoking status, pack-years, BMI, hypertension, diabetes, and urinary creatinine), and other metals that were included in the corresponding multiple-metal models. Interaction p-values represent the p-value for the interaction term. All stratified models were adjusted for other confounders and metals included in the corresponding multiple-metal models.

*Non-smokers include never and ex-smokers. Data were presented as estimated percent changes in HRV parameters associated with a ten-fold increase in each urinary metal.

Table S6. Percent difference (95% CI) in HRV parameters according to BMI, hypertension and hyperlipidemia.

Variable	BMI (< 25 kg/m^2, n=1260)	BMI (≥ 25 kg/m^2, n=744)	*p* for interaction	Subjects without hypertension (n=1283)	Subjects with hypertension (n=721)	*p* for interaction	Subjects without hyperlipidemia (n=1168)	Subjects with hyperlipidemia (n=836)	*p* for interaction
**SDNN**									
Titanium	7.48 (1.09, 14.26)	4.97 (-3.28, 13.92)	0.841	6.22 (0.07, 12.75)	8.17 (-0.58, 17.70)	0.833	2.40 (-3.97, 9.19)	11.64 (3.51, 20.39)	0.225
Copper	-7.58 (-14.62, 0.04)	-9.50 (-19.22, 1.40)	0.803	-8.76 (-15.76, -1.19)	-8.71 (-18.23, 1.92)	0.669	-5.24 (-12.99, 3.21)	-10.29 (-18.78, -0.92)	0.499
**r-MSSD**									
Titanium	12.84 (4.54, 21.79)	1.68 (-7.87, 12.23)	0.554	6.11 (-1.41, 14.19)	11.89 (0.82, 24.16)	0.373	7.64 (-0.62, 16.59)	7.07 (-2.42, 17.48)	0.812
Cadmium	-9.74 (-17.48, -1.26)	-0.94 (-11.85, 11.32)	0.049	-8.56 (-16.03, -0.42)	2.17 (-9.88, 15.82)	0.054	-5.22 (-13.42, 3.76)	-5.23 (-15.52, 6.32)	0.371
Lead	-8.15 (-14.87, -0.91)	-8.37 (-17.77, 2.12)	0.600	-7.05 (-13.57, -0.04)	-12.04 (-21.69, -1.21)	0.974	-10.25 (-17.00, -2.96)	-5.05 (-14.32, 5.22)	0.326
**Low frequency**									
Titanium	31.87 (10.52, 57.34)	11.93 (-11.73, 41.94)	0.099	20.71 (1.63, 43.37)	33.57 (4.33, 71.02)	0.958	16.33 (-2.76, 39.18)	33.17 (5.89, 67.48)	0.657
Arsenic	-15.82 (-33.16, 6.01)	-28.06 (-50.03, 3.56)	0.145	-22.12 (-37.98, -2.21)	-19.23 (-44.07, 16.64)	0.829	-19.42 (-37.18, 3.37)	-20.92 (-42.00, 7.82)	0.900
Rubidium	28.99 (-1.35, 68.67)	13.16 (-22.91, 66.11)	0.183	24.13 (-3.65, 59.92)	21.43 (-20.40, 85.22)	0.655	25.01 (-4.60, 63.80)	18.83 (-17.77, 71.73)	0.944
Cadmium	-29.04 (-43.54, -10.82)	-0.37 (-26.51, 35.09)	0.863	-13.71 (-30.60, 7.28)	-25.18 (-46.33, 4.30)	0.500	-21.57 (-37.31, -1.88)	-11.57 (-35.11, 20.51)	0.647
**High frequency**									
Titanium	40.28 (15.52, 70.33)	18.49 (-8.79, 53.92)	0.472	26.64 (4.97, 52.79)	38.69 (5.49, 82.34)	0.905	25.67 (2.17, 54.57)	34.11 (5.48, 70.51)	0.711
Iron	-9.31 (-22.01, 5.44)	-17.14 (-32.99, 2.47)	0.545	-12.66 (-24.46, 0.97)	-10.88 (-28.82, 11.59)	0.989	-10.47 (-23.50, 4.79)	-15.06 (-30.32, 3.54)	0.867
Cadmium	-24.61 (-40.04, -5.20)	-9.85 (-33.85, 22.87)	0.401	-19.22 (-35.10, 0.54)	-7.11 (-33.19, 29.15)	0.571	-20.75 (-37.31, 0.19)	-9.67 (-32.95, 21.71)	0.520
Lead	-11.61 (-27.42, 7.65)	-18.48 (-39.03, 8.98)	0.631	-5.48 (-21.76, 14.20)	-32.36 (-50.39, -7.77)	0.114	-14.35 (-30.22, 5.13)	-13.08 (-33.75, 14.05)	0.908
**Total power**									
Titanium	30.50 (12.76, 51.04)	14.30 (-5.75, 38.60)	0.398	18.76 (3.28, 36.56)	35.77 (10.54, 66.76)	0.571	14.79 (-1.28, 33.48)	37.02 (14.09, 64.56)	0.241
Cadmium	13.42 (-27.07, 2.80)	-9.35 (-27.82, 13.85)	0.835	-13.14 (-26.14, 2.14)	-5.29 (-26.07, 21.33)	0.722	-8.46 (-22.84, 8.60)	-15.12 (-32.35, 6.50)	0.899
Lead	-13.85 (-25.50, -0.38)	-10.67 (-27.71, 10.38)	0.881	-9.54 (-21.22, 3.87)	-20.11 (-36.48, 0.49)	0.384	-16.25 (-27.74, -2.94)	-3.92 (-21.54, 17.67)	0.365

Abbreviations: SDNN, standard deviation of the normal-to-normal intervals; r-MSSD, square root of the mean squared difference between adjacent normal-to-normal intervals. Each interaction model included one metal-risk factor interaction term and covariates (age, gender, smoking status, pack-years, BMI, hypertension, diabetes, and urinary creatinine), and other metals that were included in the corresponding multiple-metal models. Interaction p-values represent the p-value for the interaction term. All stratified models were adjusted for other confounders and metals included in the corresponding multiple-metal models. Data were presented as estimated percent changes in HRV parameters associated with a ten-fold increase in each urinary metal.

Table S7. Percent difference (95% CI) in HRV parameters according to diabetes and FRS.

Variable	Subjects without diabetes (n=1840)	Subjects with diabetes (n=164)	*p* for interaction	Subjects with low-risk FRS (≤ 5%, n=960)	Subjects with high-risk FRS (> 5%, n=835)	*p* for interaction
**SDNN**						
Titanium	6.56 (1.36, 12.04)	12.96 (-9.27, 40.63)	0.225	2.00 (-4.83, 9.32)	13.79 (4.72, 23.65)	0.195
Copper	-6.49 (-12.51, -0.05)	-24.30 (-41.74, -1.63)	0.046	-12.32 (-20.08, -3.82)	-10.43 (-19.49, -0.34)	0.813
**r-MSSD**						
Titanium	8.25 (1.65, 15.27)	6.69 (-15.61, 34.88)	0.586	3.20 (-4.96, 12.05)	13.65 (3.15, 25.21)	0.131
Cadmium	-5.73 (-12.45, 1.50)	-12.00 (-32.30, 14.40)	0.483	-14.06 (-21.28, -6.19)	-0.93 (-11.50, 1.90)	0.127
Lead	-7.99 (-13.78, -1.82)	-10.96 (-28.81, 11.36)	0.504	-4.07 (-11.33, 3.79)	-11.59 (-20.71, -1.43)	0.747
**Low frequency**						
Titanium	19.97 (3.66, 38.84)	116.66 (20.43, 289.80)	0.271	26.17 (1.60, 56.69)	47.69 (17.37, 85.83)	0.858
Arsenic	-15.67 (-30.91, 2.93)	-59.39 (-82.92, -3.49)	0.085	-35.26 (-50.67, -15.02)	-39.88 (-56.98, -15.97)	0.257
Rubidium	20.33 (-3.85, 50.60)	36.68 (-49.11, 267.07)	0.536	51.75 (10.85, 107.74)	41.59 (-3.50, 107.75)	0.299
Cadmium	-17.73 (-31.78, -0.78)	-25.52 (-64.99, 58.45)	0.557	-38.81 (-52.43, -21.28)	-37.21 (-53.18, -15.81)	0.429
**High frequency**						
Titanium	30.92 (11.33, 53.95)	41.45 (-21.95, 156.35)	0.555	23.44 (-1.16, 54.18)	37.89 (7.60, 76.71)	0.411
Iron	-9.51 (-20.39, 2.85)	-38.33 (-60.45, -3.83)	0.072	-4.80 (-19.34, 12.37)	-26.94 (-41.00, -9.54)	0.065
Cadmium	-19.05 (-33.15, -1.99)	-9.23 (-53.35, 76.60)	0.741	-36.06 (-49.59, -18.89)	-7.60 (-30.82, 23.42)	0.176
Lead	-11.48 (-25.33, 4.95)	-33.65 (-62.98, 18.91)	0.120	-3.73 (-22.55, 19.66)	-16.50 (37.07, 10.81)	0.593
**Total power**						
Titanium	23.77 (9.77, 39.55)	48.26 (-8.99, 141.52)	0.718	19.59 (1.42, 41.01)	41.78 (16.60, 72.39)	0.416
Cadmium	-11.54 (-23.18, 1.88)	-25.72 (-56.97, 28.22)	0.843	-28.84 (-40.30, -15.19)	-22.78 (-38.49, -3.05)	0.937
Lead	-12.51 (-22.72, -0.96)	-7.99 (-42.24, 46.58)	0.951	-8.37 (-21.73, 7.28)	-8.51 (-26.54, 13.93)	0.958

Abbreviations: FRS, Framingham risk score; SDNN, standard deviation of the normal-to-normal intervals; r-MSSD, square root of the mean squared difference between adjacent normal-to-normal intervals. Each interaction model included one metal-risk factor interaction term and covariates (age, gender, smoking status, pack-years, BMI, hypertension, diabetes, and urinary creatinine), and other metals that were included in the corresponding multiple-metal models. Interaction p-values represent the p-value for the interaction term. All stratified models were adjusted for other confounders and metals included in the corresponding multiple-metal models. Data were presented as estimated percent changes in HRV parameters associated with a ten-fold increase in each urinary metal.

Figure S1. Distribution of natural log-transformed HRV parameters. Abbreviations: lnSDNN, natural log-transformed standard deviation of the normal-to-normal intervals; lnr-MSSD, natural log-transformed square root of the mean squared difference between adjacent normal-to-normal intervals; lnLF, natural log-transformed low frequency; lnHF, natural log-transformed high frequency; lnTP, natural log-transformed total power. p-values were derived from Kolmogorov-Smimov test.

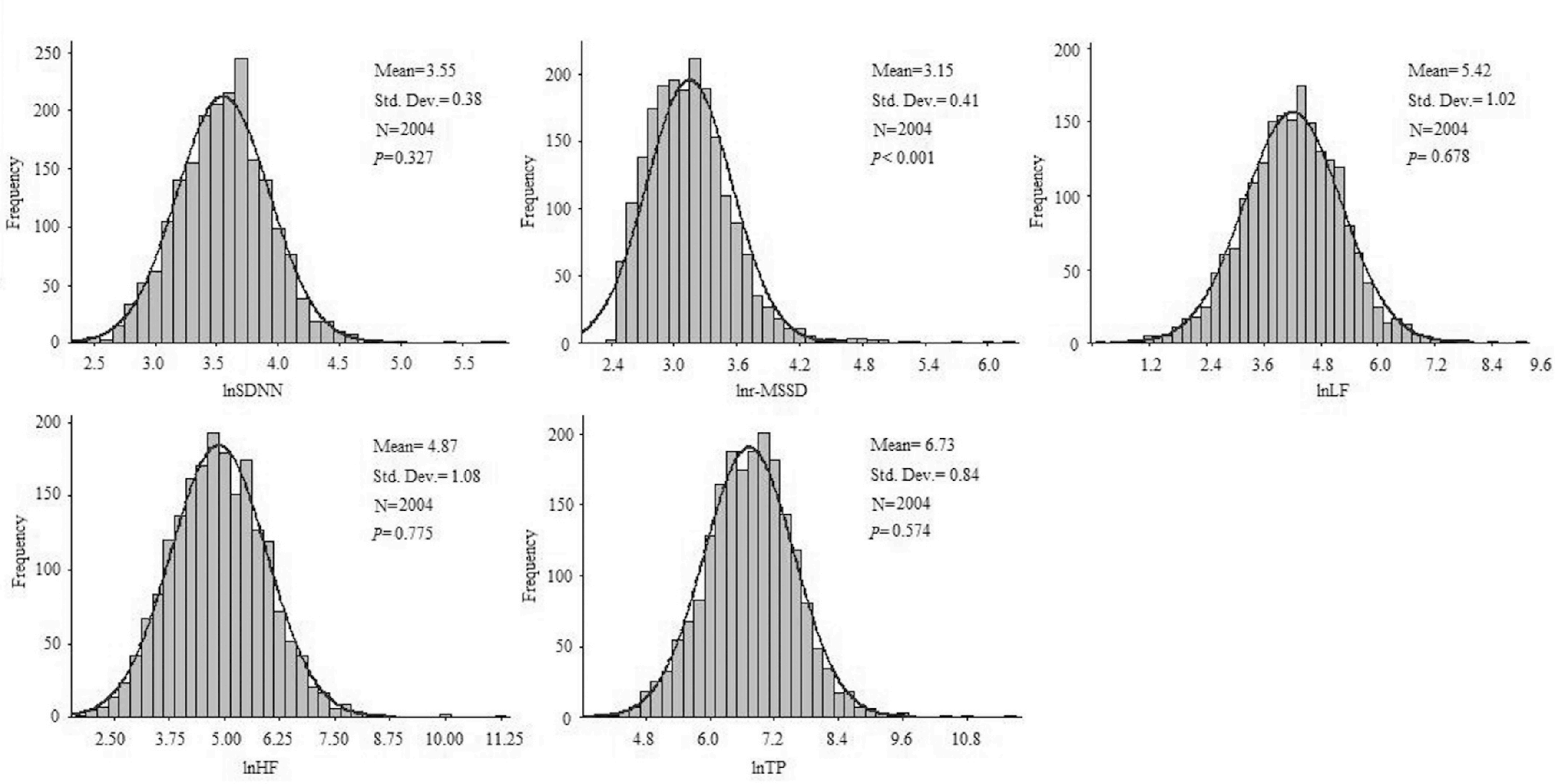

Figure S2. Estimated percent difference (95%CI) in HRV indices with a 10-fold increase in creatinine-standardized urine metal concentrations based on single-metal linear regression models adjusted for age, gender, smoking status, pack-years, BMI, hypertension, hyperlipidemia and diabetes. *FDR-adjusted $p < 0.05$. Abbreviations: FDR, false discovery rate; SDNN, standard deviation of the normal-to-normal intervals; r-MSSD, square root of the mean squared difference between adjacent normal-to-normal intervals.

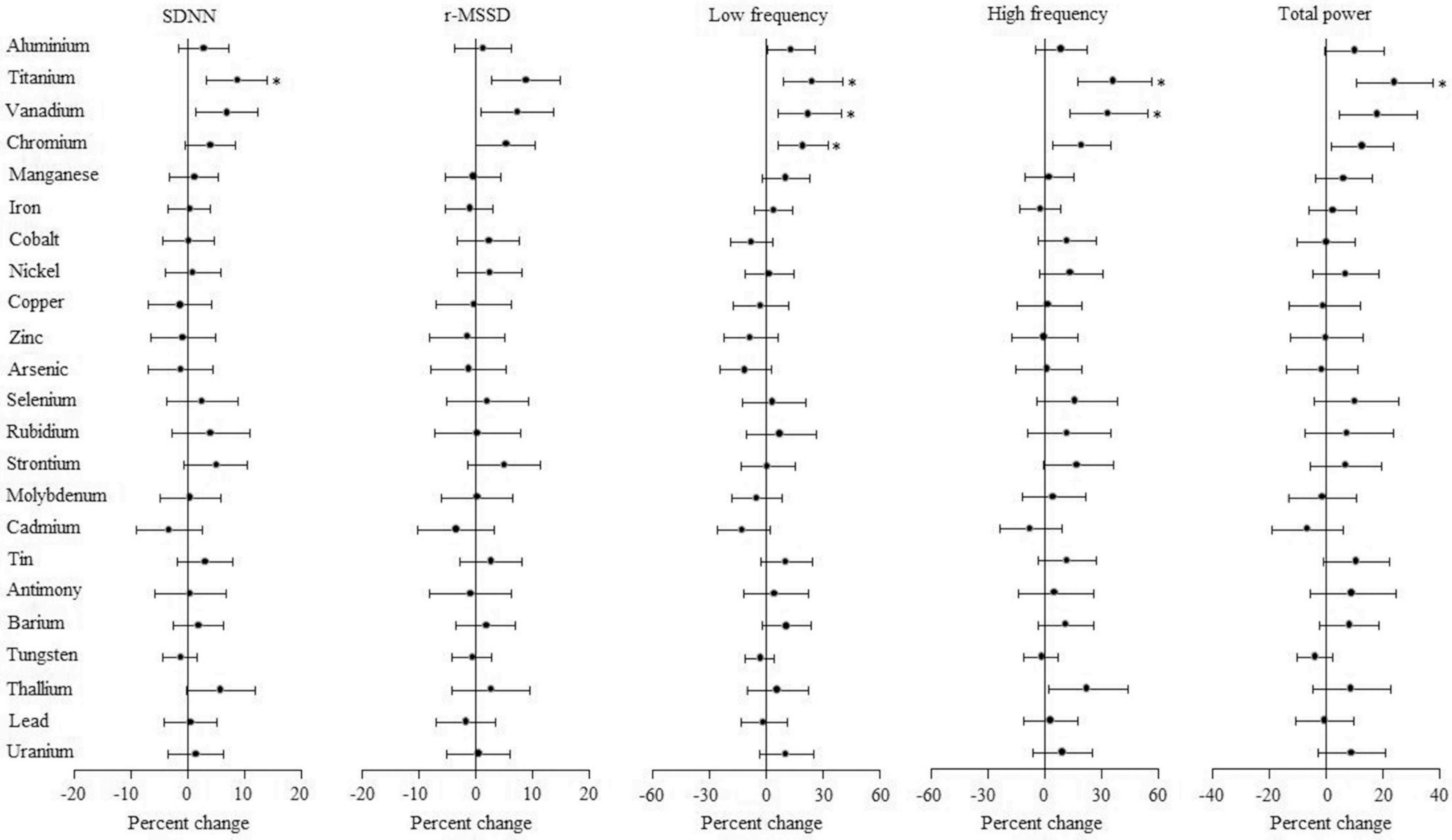

Supplement: (1.3 MB) PDF [file ehp.1307563.s001.508.pdf]
